# Twenty-one years of cutaneous melanoma: stage shift, anatomical patterns and diagnostic trends from a Southern European tertiary center

**DOI:** 10.1097/CMR.0000000000001115

**Published:** 2026-06-12

**Authors:** Nello Tommasino, Claudia Costa, Alessia Villani, Luca Menna, Adriana Di Guida, Luigi Coronella, Maddalena Napolitano, Massimiliano Scalvenzi

**Affiliations:** Section of Dermatology, Department of Clinical Medicine and Surgery, University of Naples Federico II, Naples, Italy

**Keywords:** Breslow thickness, dermoscopy, early detection, epidemiology, melanoma

## Abstract

Cutaneous melanoma incidence continues to rise worldwide, while advances in dermoscopic surveillance increasingly influence the stage at diagnosis. Long-term single-center analyses offer a unique perspective on how demographic shifts, tumor biology, and diagnostic intensity interact over time. We retrospectively analyzed 4062 consecutive patients with histologically confirmed cutaneous melanoma diagnosed between 2003 and 2024 at a tertiary dermatology center in Southern Italy. Demographic characteristics, anatomical site, and Breslow thickness were collected. Temporal trends were assessed using regression models and *χ*^2^ tests for trend. Melanoma diagnoses increased significantly over the study period (*P* < 0.0001), with a near three-fold rise between the first (2003–2013) and second (2014–2024) decades. In parallel, the proportion of melanoma in situ increased from 38.2 to 57.5% (*P* < 0.0001), indicating a pronounced shift toward earlier-stage detection. Mean age at diagnosis rose significantly over time (*P* < 0.001). Trunk melanomas predominated overall, particularly among men, whereas lower limb melanomas were more frequent among women (*P* < 0.001). Acral melanomas, although uncommon, showed the highest proportion of thick lesions (48.6%; ≥0.8 mm). Over two decades, melanoma diagnoses increased markedly alongside a substantial shift toward early stage disease. While these trends reflect improvements in surveillance and diagnostic sensitivity, the persistent burden of thick melanomas – particularly in acral locations and older patients – underscores unresolved diagnostic gaps.

## Graphical abstract

**Figure:**
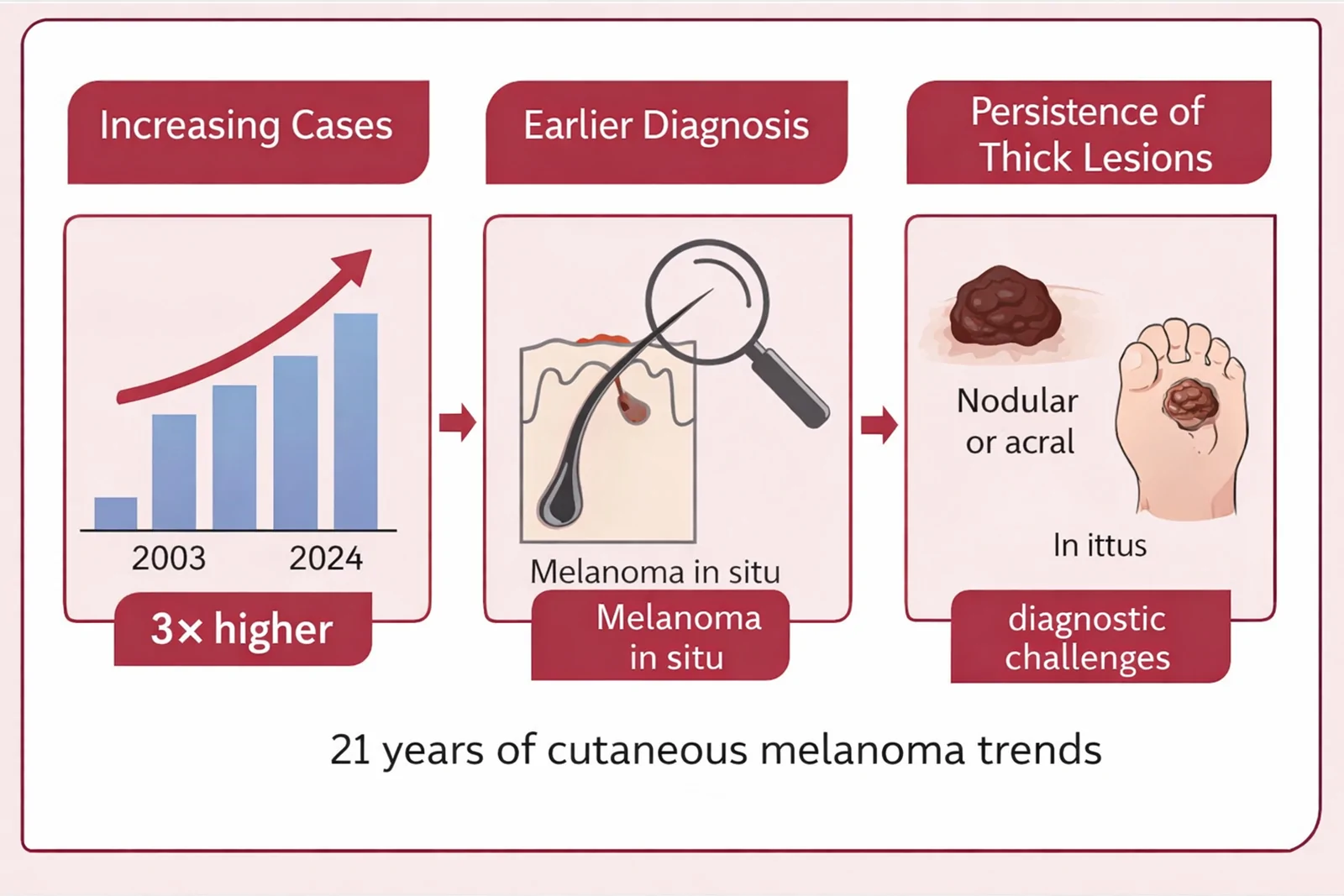

## Introduction

Cutaneous melanoma arises from melanocytic cells and represents a growing public health issue in many high-income regions. Worldwide estimates indicate over 200 000 new cases annually across sexes, with notable geographic variation influenced by ultraviolet (UV) exposure patterns and population phototypes [1,2]. Incidence has risen since the 1970s, and although younger cohorts show some stabilization, overall rates continue to climb [3]. Established risk factors include intermittent intense UV exposure, host phenotype (fair skin, freckles, light hair, and eye color), high nevus count, and family history of melanoma [4–6]. Dermoscopy and structured skin surveillance have progressively improved diagnostic sensitivity for early stage lesions, increasing the detection of melanoma in situ and thin invasive tumors [7,8]. However, population subgroups continue to present with late-stage disease, with implications for prognosis and health services. Long-term single-center series provide insights into temporal changes driven by diagnostics, public health interventions, and demographic shifts. The present study analyzes 4062 melanoma cases diagnosed between 2003 and 2024 at the Dermatology Unit of the University of Naples Federico II to characterize trends in age, sex, anatomical distribution, and Breslow thickness and to inform local prevention priorities.

## Materials and methods

We retrospectively reviewed the institutional database and histopathology reports to identify all consecutive patients with a primary cutaneous melanoma diagnosed from January 2003 to December 2024. Inclusion criteria were histological confirmation of melanoma (including melanoma in situ, superficial spreading melanoma, and nodular melanoma) and availability of data on sex, age at diagnosis, primary anatomical site, and Breslow thickness. Exclusion criteria were noncutaneous melanomas, melanomas arising in mucosal or ocular sites, and cases with incomplete records. Demographic variables included sex and age grouped as: less than 18, 18–30, 31–40, 41–50, 51–60, 61–70 and greater than 70 years. Anatomical sites were categorized as head–neck, trunk, upper limbs, lower limbs, and acral locations. Breslow thickness was categorized as melanoma in situ, less than 0.8 mm, and greater than or equal to 0.8 mm, consistent with institutional practice and sentinel node biopsy thresholds [9]. Initial diagnostic work-up involved clinical and dermoscopic examination; excisional biopsy with narrow margins was performed when feasible, and diagnostic histology was obtained according to standard protocols. Surgical management, sentinel lymph node biopsy, and follow-up adhered to national AIOM recommendations and AJCC 8th edition staging [1,10]. We summarized case counts and proportions annually and compared two decades (2003–2013 vs. 2014–2024). Continuous variables were summarized as mean ± SD, while categorical variables were expressed as absolute counts and percentages. Differences in categorical distributions (sex, anatomical site, and Breslow thickness categories) were assessed using the *χ*^2^ test or Fisher’s exact test where appropriate. Temporal trends in categorical variables, particularly the proportion of melanoma in situ over time, were evaluated using the Cochran–Armitage *χ*^2^ test for trend. Annual melanoma incidence was modeled using Poisson regression, with calendar year as a continuous predictor, to assess changes in case numbers over time. Linear regression analysis was applied to evaluate trends in mean age at diagnosis across the study period. Comparisons between decades (2003–2013 vs. 2014–2024) were performed using *χ*^2^ tests for categorical variables and Student’s *t*-test for continuous variables. All tests were two-sided, and a *P* value less than 0.05 was considered statistically significant. Statistical analyses were conducted using standard statistical software.

## Results

A total of 4062 patients satisfied inclusion criteria. Overall, 2236 (55.0%) were male and 1826 (45.0%) female (male:female ratio: 1.22) (Fig. 1). Mean age at diagnosis was 56.53 ± 15.99 years. Age-group distribution showed the highest representation in the 61–70-year bracket (21.98%), followed by 51–60 years (21.34%) and greater than 70 years (21.29%). Linear regression analysis demonstrated a significant increase in mean age at diagnosis over time (*P* < 0.001), rising from a mean of 48.5 years in the early 2000s to approximately 60 years in the most recent decade. Pediatric occurrences were rare (0.24% of the cohort). The anatomical distribution of melanoma across the cohort revealed a clear predominance of trunk lesions, with 2387 cases (58.78%), while lower limbs had 762 (18.76%), upper limbs 480 (11.81%), head–neck 326 (8.02%), and acral sites 107 (2.63%) (Fig. 2). These represented only a small fraction of cases but remained clinically noteworthy because of their distinctive behavior and diagnostic challenges. A strong association between sex and anatomical site was observed (*P* < 0.001), with trunk melanomas predominating among men (68.3%) and lower limb melanomas disproportionately represented among women (30.2%), consistent with known sex-related behavioral and photobiological differences.

**Fig. 1:**
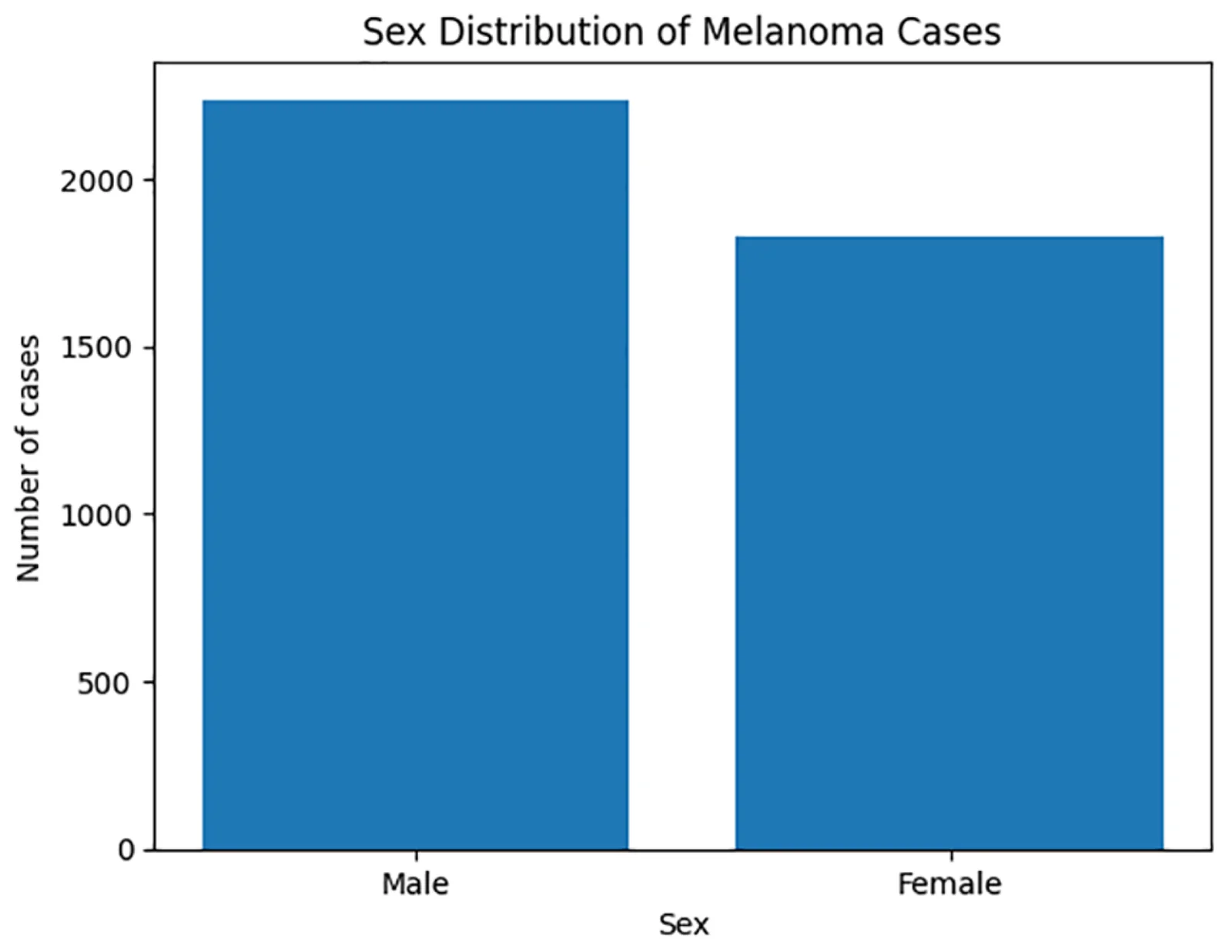
Proportion of male and female patients diagnosed with cutaneous melanoma over the 21-year study period, illustrating the overall male predominance within the cohort.

**Fig. 2:**
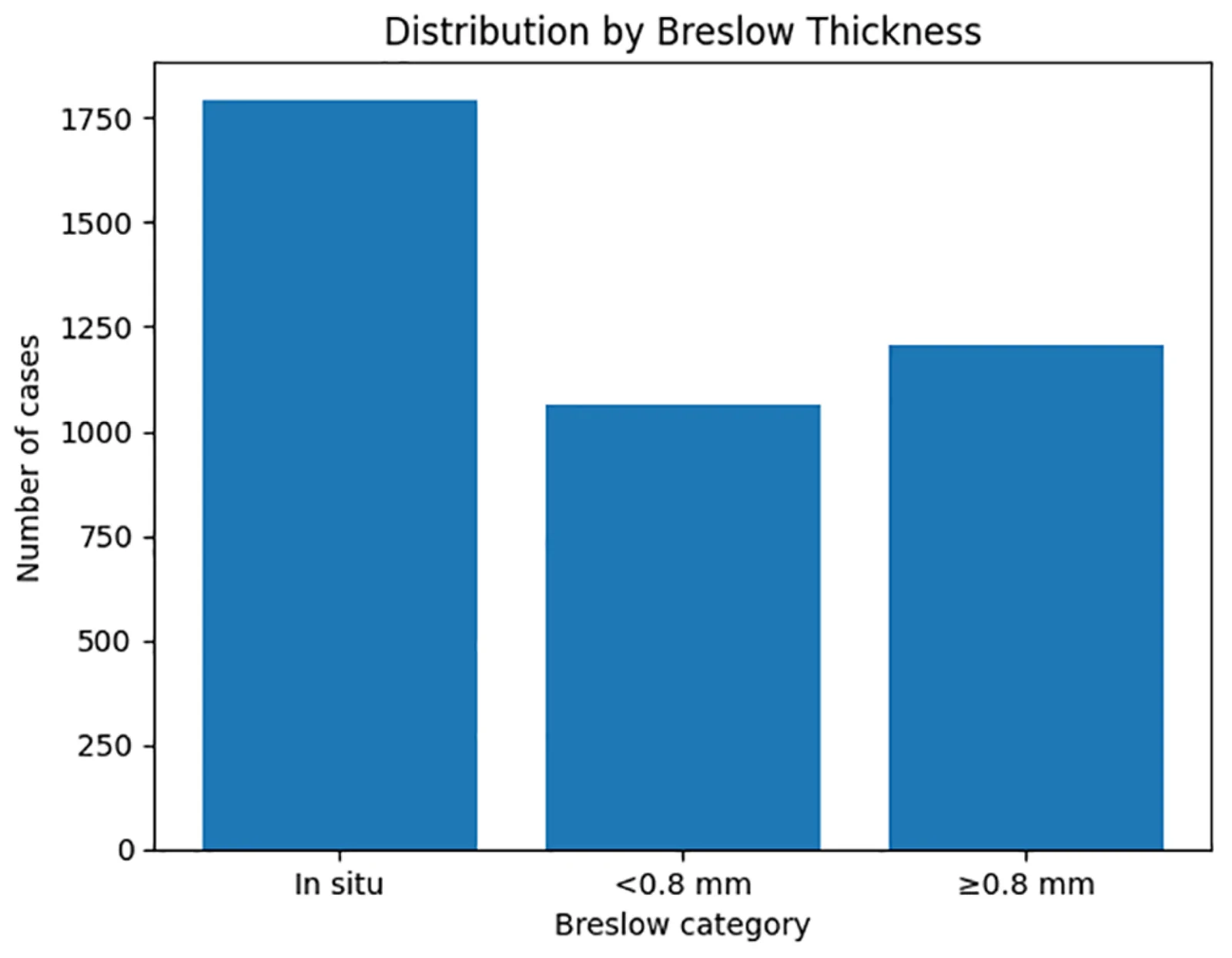
Relative frequency of melanoma by anatomical location, showing a marked predominance of trunk melanomas and distinct sex-related distribution patterns across body sites.

Breslow thickness distribution differed significantly by anatomical site (*P* < 0.0001). Across the entire cohort, melanomas in situ represented the largest proportion of diagnoses with 1794 cases (44.17%), followed by 1064 (26.19%) thin invasive melanomas (<0.8 mm), and then 1204 (29.64%) thick lesions (≥0.8 mm) (Fig. 3). Although the distribution of Breslow categories was broadly similar between men and women, females exhibited a slightly higher percentage of thick melanomas. Thickness patterns varied substantially by anatomical location: head–neck melanomas were more frequently in situ than melanomas arising elsewhere, while lower limb melanomas showed a comparatively greater proportion of thick tumors. Acral sites, despite representing the least common location, demonstrated the highest prevalence of thick melanomas, with almost half of all acral tumors exceeding 0.8 mm at diagnosis (48.6%). This finding highlights the persistent diagnostic delays associated with acral melanoma, which often presents subtle or nonspecific clinical features.

**Fig. 3:**
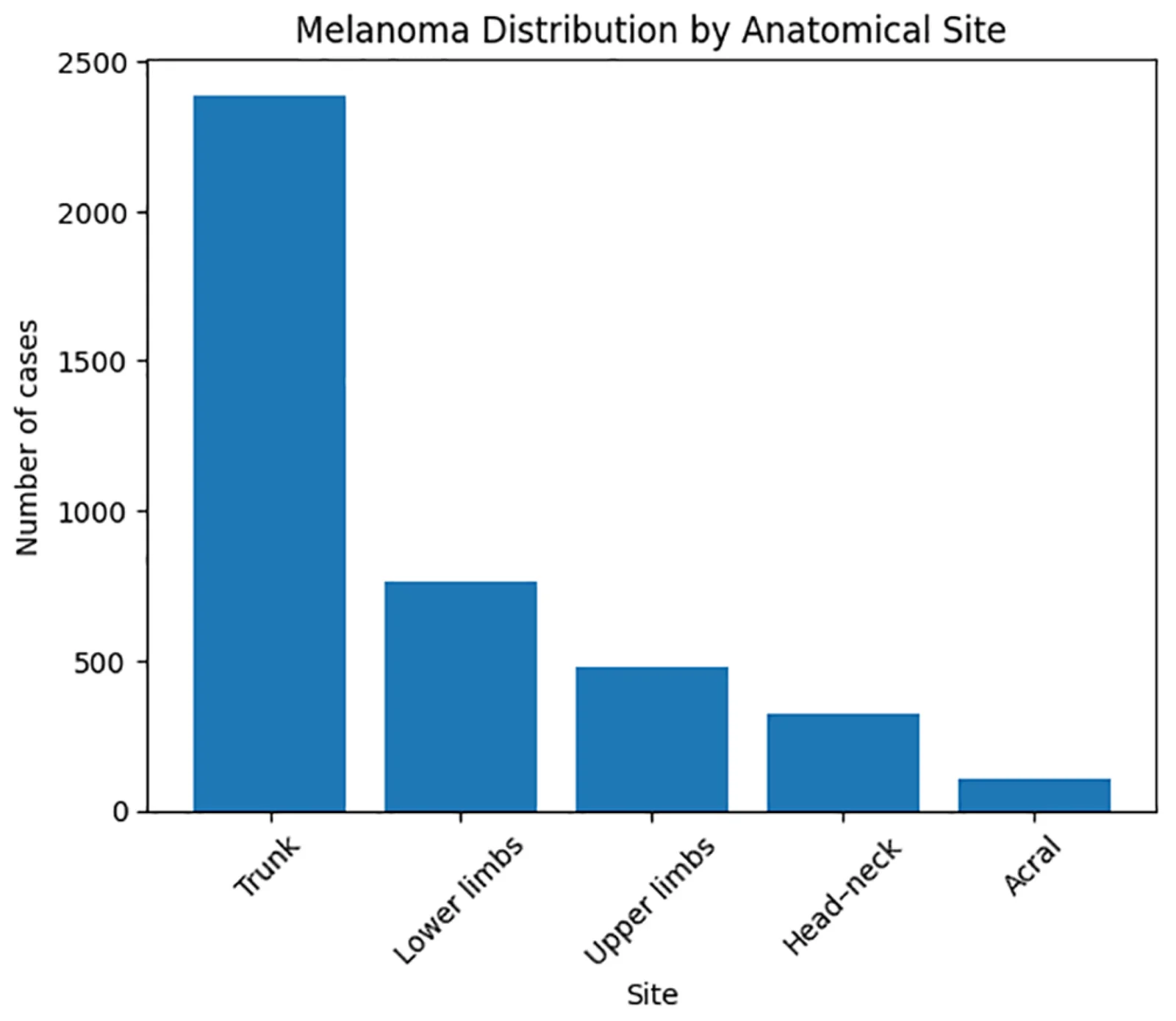
Proportion of melanoma in situ, thin invasive (<0.8 mm) and thick (≥0.8 mm) melanomas in the overall cohort, highlighting the coexistence of a substantial early stage burden with a persistent fraction of thick tumors.

Over the 21-year study period, melanoma incidence increased dramatically, from 34 cases in 2003 to 435 cases in 2024. Comparing decades, 2003–2013 included 1089 cases, while 2014–2024 included 2973 cases (Fig. 4), with a highly significant upward trend in melanoma diagnoses over this time confirmed by Poisson regression (*P* < 0.0001).

**Fig. 4:**
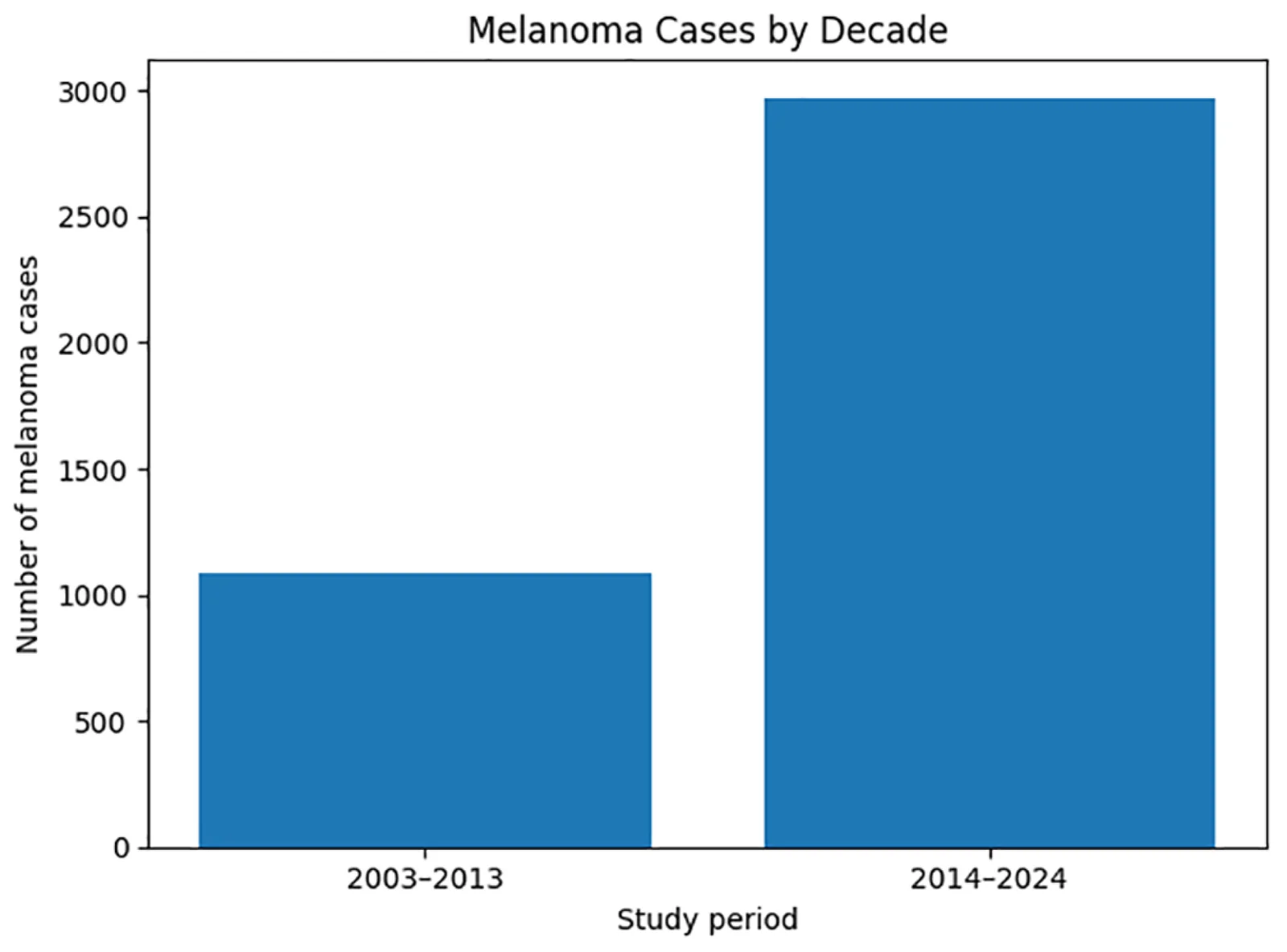
Melanoma cases incidence by decade.

In parallel, the mean age at diagnosis demonstrated a gradual but consistent upward shift. In the early 2000s, patients were diagnosed at an average age of 48.5 years; however, from 2010 onward, the average age began to rise, reaching 60.3 by 2021 and stabilizing just below this level in recent years. Although year-to-year fluctuations were observed, the overall pattern suggests a progressive aging of the melanoma population, which may reflect demographic changes, cumulative UV exposure effects, and enhanced longevity in the general population.

A particularly meaningful temporal shift emerged in the distribution of Breslow thickness over time. The proportion of melanoma in situ increased from 38.2 in 2003 to 57.5% in 2024, indicating a significant upward shift in early stage detection across the observation period (*P* < 0.0001) (Fig. 5). From 2015 onward, however, the proportion of in situ lesions began to rise steadily and markedly, surpassing 55% in several of the most recent years and reaching its highest recorded value in 2024. Although the absolute number of thick melanomas grew in parallel with the overall rise in diagnoses, their relative frequency decreased when compared to the rapidly expanding number of melanomas in situ. The divergence of these trends indicates a meaningful shift in clinical practice: more lesions are being identified at an earlier stage, while late-stage presentations, though still present, now constitute a smaller proportion of the melanoma burden.

**Fig. 5:**
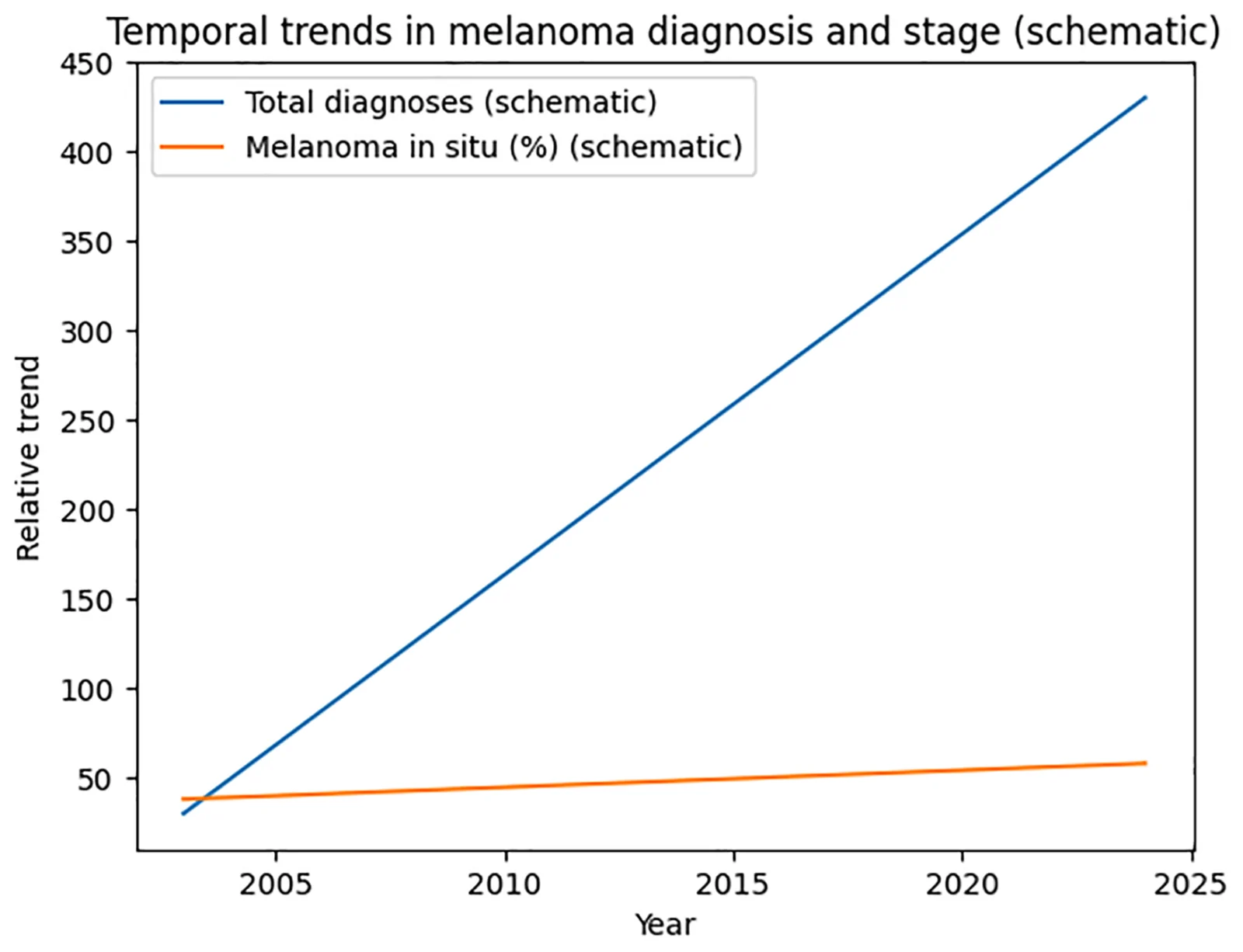
Temporal trends in melanoma diagnoses and stage at presentation. It illustrates the parallel increase in melanoma diagnoses and the progressive shift toward in situ disease.

## Discussion

This 21-year retrospective study provides one of the most extensive epidemiologic analyses of melanoma in Southern Italy, offering valuable insights into temporal trends, demographic shifts, and evolving patterns of early detection. It confirms several international observations while providing local details relevant for clinical practice and public health planning.

The marked rise in absolute melanoma diagnoses parallels global patterns and likely reflects a combination of true increases in incidence and improved case ascertainment [11]. The implementation and routine use of dermoscopy in the early 2000s revolutionized cutaneous oncology, improving diagnostic accuracy and increasing clinician sensitivity to subtle melanoma signs [12]. Concurrently, heightened public awareness and increased frequency of dermatologic evaluation contributed to the enhanced detection of early stage lesions [7,8]. Indeed, the contemporaneous and pronounced rise in melanoma in situ proportion strongly suggests that enhanced early detection, rather than purely biological shifts, has played a major role. The increase of in situ detection from 38 to 57% over two decades likely reflects improved clinician expertise, routine dermoscopic monitoring, and possibly screening efforts targeted at patients with multiple or atypical nevi. The surge was particularly pronounced after 2015, coinciding with greater availability of digital dermoscopy and structured surveillance systems within the regional healthcare network. This pattern parallels observations from several high-income countries where early detection campaigns have led to increasing proportions of thin or in situ melanomas. Early excision of suspicious lesions limits progression and may reduce melanoma-specific mortality at the population level, although robust evidence for mortality reduction remains complex because of lead-time and overdiagnosis considerations [13].

The demographic shifts observed – particularly aging of the diagnosis cohort – require careful interpretation. Our data indicate a progressive rise in mean age at diagnosis (from mid-late 40s to near 60s), which may be influenced by multiple factors, including demographic aging of the catchment population, cumulative lifetime UV exposure, and improved survival from other causes leading to a larger elderly population undergoing dermatologic surveillance. An aging presentation has implications for management, as older patients more frequently harbor comorbidities that complicate surgical and systemic treatment decisions [14]. Tailored care pathways and geriatric assessment could enhance outcome optimization in this subgroup.

The sex distribution observed in our study (male:female ratio: 1.22) aligns with international epidemiologic observations and reflects complex interactions between behavioral patterns, photoprotection habits, and possibly biological factors [15]. Women tended to receive diagnoses at younger ages, particularly between 41 and 50 years, whereas men were disproportionately represented in older age brackets [16]. These findings reinforce the concept that men often engage less in preventive care, perform fewer self-examinations, and may delay seeking medical evaluation. In addition, sex-based anatomical patterns observed in our cohort are largely consistent with established behavioral models. Men demonstrated a striking predominance of trunk melanomas, while women displayed a substantial burden on lower limbs [17]. The higher frequency of trunk lesions in men is often attributed to intermittent, intense sun exposure during leisure activities and reduced routine skin self-examination, whereas women’s higher lower limb incidence may relate to clothing habits and tanning behaviors [17,18]. These sex-specific distributions underscore the need for targeted public health messages: for example, reinforcing trunk-focused surveillance in men and encouraging regular lower limb checks in women.

Acral melanomas, while constituting a small fraction of diagnoses, remain disproportionately represented among thick tumors, with nearly half presenting at greater than or equal to 0.8 mm. This observation is consistent with established literature emphasizing diagnostic delay and atypical clinical presentation of acral lesions [19]. Clinicians should maintain a high index of suspicion for nonpigmented or atypically pigmented acral lesions and consider earlier biopsy for persistent or evolving lesions in these sites.

The relative decline in thick melanomas and increase in thin tumors indicate progress in early diagnosis, but the persistent proportion of thick lesions (nearly 30% overall) highlights ongoing diagnostic gaps. These gaps may reflect disparities in access to dermatologic services, lower health literacy in specific subpopulations (e.g. older men), or biological differences leading to more aggressive tumor growth in certain subtypes (e.g. nodular melanoma). Notably, nodular and amelanotic melanomas are often thicker at diagnosis and can evade early dermoscopic detection, necessitating continued refinement of diagnostic algorithms and clinician education [20,21].

Limitations of our study include its retrospective, single-center design, which may limit generalizability; potential referral bias toward more severe or diagnostically challenging cases; and the absence of outcome data such as recurrence and survival, which would enrich prognostic interpretation. Strengths include the large sample size, extended observation period, and consistent diagnostic and management pathways. Future work should integrate stage-specific outcomes, molecular profiling (including BRAF and other driver mutations), and socioeconomic determinants to better elucidate drivers of late presentation and to design tailored interventions.

In practical terms, our findings support several actionable recommendations: maintain and expand dermoscopy training for primary care physicians and dermatologists; implement targeted outreach campaigns focused on older males and on recognizing acral and amelanotic presentations; integrate teledermoscopy and triage systems to improve access in underserved areas; and promote lifelong skin surveillance for patients with high nevus counts or personal/family melanoma history. Collectively, these strategies may further shift the diagnostic distribution toward earlier-stage melanomas and ultimately improve patient-centered outcomes.

Across two decades at a tertiary dermatology center in Southern Italy, melanoma diagnoses increased markedly while the proportion of early stage (in situ) disease rose substantially. These trends indicate improved detection and surveillance but coexist with a persistent fraction of thick tumors, highlighting subgroups that remain vulnerable to delayed diagnosis. Targeted public health efforts, clinician education, and systematic surveillance – particularly aimed at older men and at lesions in acral locations – are recommended to further reduce the burden of advanced melanoma.

## Acknowledgements

We would like to thank everyone who contributed to the completion of this work.

Conceptualization: N.T., C.C., L.M., and M.S. Methodology: N.T., A.V., L.M., and M.N. Formal analysis and investigation: N.T., C.C., A.V., A.D.G., and M.S. Writing – review and editing: N.T., L.M., L.C., and M.S. Supervision: C.C., A.V., M.N., and M.S. All the authors read and approved the final version of the manuscript and agreed to be accountable for all aspects of the work.

This study protocol was reviewed and approved by ‘Campania 3 Ethics Committee’.

The data supporting the findings of this study are available from the corresponding author upon reasonable request. Because of ethical and privacy restrictions, individual-level patient data are not publicly available.

### Conflicts of interest

There are no conflicts of interest.
